# Predicting the Distribution of Spiral Waves from Cell Properties in a Developmental-Path Model of *Dictyostelium* Pattern Formation

**DOI:** 10.1371/journal.pcbi.1000422

**Published:** 2009-07-10

**Authors:** Daniel Geberth, Marc-Thorsten Hütt

**Affiliations:** Computational Systems Biology, School of Engineering and Science, Jacobs University Bremen, Bremen, Germany; University of Washington, United States of America

## Abstract

The slime mold *Dictyostelium discoideum* is one of the model systems of biological pattern formation. One of the most successful answers to the challenge of establishing a spiral wave pattern in a colony of homogeneously distributed *D. discoideum* cells has been the suggestion of a developmental path the cells follow (Lauzeral and coworkers). This is a well-defined change in properties each cell undergoes on a longer time scale than the typical dynamics of the cell. Here we show that this concept leads to an inhomogeneous and systematic spatial distribution of spiral waves, which can be predicted from the distribution of cells on the developmental path. We propose specific experiments for checking whether such systematics are also found in data and thus, indirectly, provide evidence of a developmental path.

## Author Summary

Spatio-temporal pattern formation is a core discipline of theoretical biology. Formation of large-scale patterns from local interactions can very prominently be observed in the swarming behavior of fish and birds, in animal markings or bacterial growth patterns. It also plays a critical role in the life cycle of the social amoeba *Dictyostelium discoideum*. A homogeneous colony of amoebae is partitioned into subgroups that will form migrating slugs by a collective phase of chemotactic signaling, exhibiting typical and well-known patterns for this sort of excitable dynamics (circular and spiral waves). The mechanism of spatial localization of aggregation centers (that is, the centers of periodic circular and spiral waves) is unclear, despite its crucial role to the organism's procreation. Here we demonstrate for an established computational model of *D. discoideum* that the initial properties of potentially very few cells have a driving influence on the resulting asymptotic collective state of the colony. Analogous processes take place in diverse situations such as, e.g., heart cells (where spiral waves occur in potentially fatal ventricular fibrillation), so that a deeper understanding of this additional layer of self-organized pattern formation would be beneficial to a wide range of applications.

## Introduction

The slime mold *Dictyostelium discoideum* is a model organism for the study of pattern formation and excitable media dynamics in biological systems [1]. Several stages of its life cycle exhibit self-organized formation of structures successively building up on one another. Here we are exclusively concerned with the starvation-induced passage from a colony of chemotactically quiescent single cells to the cAMP signaling stage prior to the onset of aggregation and slug formation. Starting from the spontaneous emissions of a few starving cells, the whole colony enters a regime of excitable media dynamics [2]–[4], where a local supra-threshold concentration of cAMP causes cells to produce and release more cAMP, which then diffuses to neighboring cells. The behavior of the single cells gives rise to a macroscopic dynamics exhibiting typical excitable media attractor states, namely circular radially growing ‘target waves’ caused by periodic oscillation of a central pacemaker element and self-sustained spiral waves. There are several mathematical models describing this transition [5]–[7], making different assumptions about the exact nature of the underlying biological processes.

Single *D. discoideum* cells have recently been shown experimentally to have distinct and persistent reactions to external stimuli [8]. In particular, the response to repeated stimuli varied substantially less for an individual cell under repeated stimuli, compared to the ensemble variation, indicating that the average response is indeed a cell property, varying across the cell population but rather fixed in time.

As an analogy to physical systems, we like to interpret the arising situation as a ‘jagged potential landscape’; a hypothetical basic process corresponds to a smooth potential, where an injected particle will almost certainly come to rest in some sink of the landscape, and, in the case of several stable conformations emerging under variation of some control parameter and separated by unstable equilibrium positions (the typical scenario for a second-order phase transition), random temporal fluctuations such as thermal noise decide the result (see, e.g. [9] for the relation between phase transitions and self-organized processes). However, the biological variability of a real system combined with the finite number of constituents adds a layer of static roughness to the potential landscape, so that the influence of small ‘bumps’ may well outrank thermal noise, leaving a distinct fingerprint of the cell configuration in an ensemble of experiments and thus systematically biasing the asymptotic configuration of the system.

We believe that in principle the result of the self-organized signaling, namely the spatial layout of the spiral wave pattern, can be predicted from the location and the properties of some cells playing key roles in triggering certain phases of cAMP communication. We recently succeeded in demonstrating this in a rather detailed fashion [10] for the model developed in [6],[11], which was also used to draw a connection between the macroscopic spiral wave density and the genetic feedback strength of the cAMP dynamics [12]. A key finding of [10] is the pronounced anticorrelation between the location of pacemaker cells (which are explicitly included in that model) and spiral occupancy, which enabled us to identify (and model geometrically) the most relevant microscopic mechanism of spiral formation, leading to a quantitatively successful prediction scheme for the spiral tip probability based only on pacemaker cell locations.

If for several mathematical models of one real system the mechanisms and rules can be identified that map single element properties to emerging patterns, one can check these for agreements and differences which may open a route to experimental testing of macroscopic predictions, thus testing the assumptions regarding microscopic processes that are not easily accessible to direct observation.

Therefore we here explore the deep link between cell properties and pattern features in a realistic mathematical model of *Dictyostelium* signaling behavior, namely the developmental path model [5], which is motivated by experimental evidence for a slow variation of the cells' kinetic properties [13]–[15].

The Methods section describes the model we analyze here, as well as giving a brief overview over the methods we used to detect significant events in the spatio-temporal evolution of the system and the analysis of point processes, which we used to relate our numerical findings to experimental data. Using these we were able to identify effective pacemaker cells and spiral creation statistics, as described in the results section, enabling us to find qualitative differences in the mapping of pacemaker positions to macroscopic spiral probabilities in two models of *D. discoideum*.

## Results

### Analysis of target and spiral wave formation

Although the model used here does not explicitly contain pacemaker cells like some other models of *D. discoideum* ([6],[12]), we can expect that, since signal transduction is enabled rather suddenly, when almost all cells have crossed into the excitable regime, only the cells that are in the oscillatory regime at that exact moment in time can be the ones to initiate the first generation of target waves. These cells effectively assume the role of pacemakers in the early shaping of the emerging patterns.

**Figure 1:**
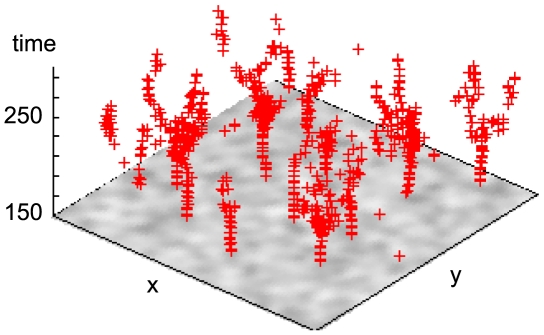
Detected target wave events (red +) over density plot of ‘pacemaker’ cells that are in the oscillatory regime when signaling begins at 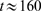. The tree-like structure of fracturing target wave emitters is clearly visible. The shown density of pacemaker cells is a Gaussian smoothing (width 2.0 grid points) of the binary distribution, color runs from white (0.0) to black (1.0).

Figure 1 shows target wave events detected with the algorithm summarized in the Methods section over a density plot of cells that are in the oscillatory regime when signaling begins at 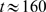. Two regimes are discernible: early locally repeating target waves growing in ‘stems’ mostly from clusters of oscillatory cells (dark areas) and a regime where several target wave centers drift apart slowly in branch-like structures, starting at about 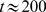, when most cells have entered the autonomously oscillatory phase of the developmental path.

In the early stage, clusters of oscillatory cells have a high chance of creating a repeating target wave center before being entrained by target waves emanating from single pacemakers in the neighborhood, since any of them can initially trigger the surrounding excitable cells; the reduced expected ‘time to next excitation’ offers a selection advantage compared to solitary oscillatory cells. The slower pacemakers of the cluster are in this case enslaved by the surrounding pattern, quickly leading to their synchronization with the initially triggering pacemaker.

**Figure 2:**
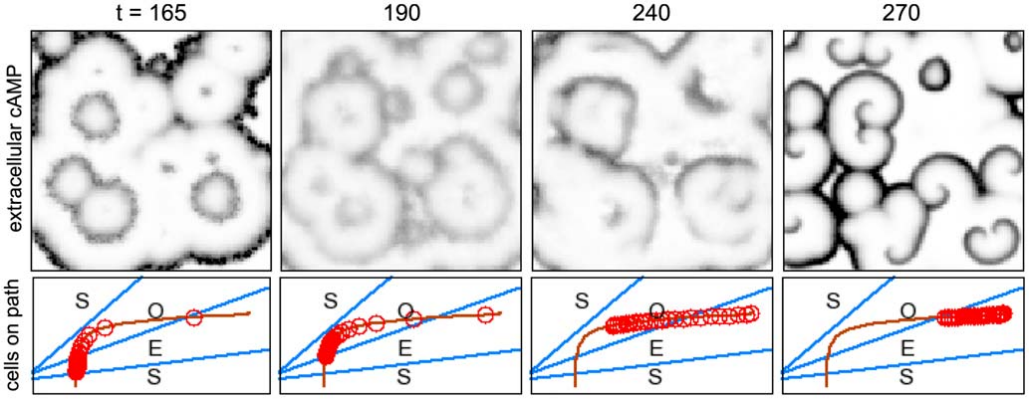
Reproduction of the results from Figure 6 in [5], using our own simulations. Top row: snapshots of the extracellular cAMP concentration 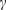. Bottom row: snapshots of the distribution of the cells on the developmental path, every circle corresponds to 4% of the cells. There is an initial quiescent period while the cells mature to become excitable. When almost all cells have become excitable, they act as a switch that activates the medium, enabling it to transmit cAMP pulses from the cells already in the oscillatory regime (leftmost column). When most cells have entered the oscillatory regime, these initially stable target waves start to fracture into smaller synchronized active centers (second column) that interact with each other and can lead to open wave ends in a typical radial distance from the original target wave center. Note that in this regime, one mainly observes dynamical synchronization, as opposed to excitable behavior. As more and more cells leave the oscillatory regime, excitable behavior visibly starts to dominate the appearance of the system again (emergence of crisp wave fronts, third and fourth column), turning the open-ended wave fronts established in the oscillatory regime into true self-sustained spiral waves. The developmental program ends with all cells back in the excitable regime, and, in the absence of cell aggregation in the model, the established spiral waves persist indefinitely (rightmost column; remnants of target waves triggered by some of the last cells to leave the oscillatory regime can still be seen).

**Figure 3:**
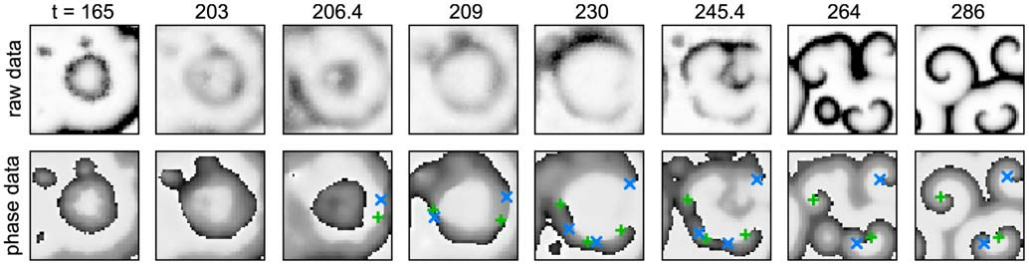
Close-up snapshots of the lower right hand corner of Figure 2, following a typical series of events leading to spiral formation. When most cells have passed into the excitable regime, target waves are initiated by the few advanced cells already in the oscillatory regime. Once more cells have crossed into the autonomously oscillatory regime, the target waves start becoming unstable and fracture into smaller active centers that synchronize dynamically, leading to open wave ends which are transformed into self-sustaining spiral waves once the cells cross back into the excitable regime. Spirals detected with the phase singularity algorithm have been superimposed (green (light gray)+for right handed and blue (dark gray)×for left handed spirals).

The behavior of predominantly oscillatory cells in the latter stage (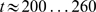) is a collective oscillatory dynamics, as opposed to the excitable behavior observed in the early and late stages of the developmental path employed here. The dynamical behavior of single cells changes qualitatively when the bulk of cells in the system crosses over into the oscillatory regime: Cells no longer react to their neighbors activating them, but oscillate autonomously. The target wave pattern established in the early phase (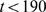) remains imprinted on the system and persists for a while, but is no longer a completely stable attractor of the collective system. The diffusive term in the extracellular cAMP concentration coupled with the degradation term act as a phase damper, favoring synchronous bulk oscillations. We believe this amplifies the irregularization of the amplitudes along the circumference of a target wave (cf. Figures 2 and 3), simplifying the breakup of waves and finally spiral formation, as described below.

Depending on the desynchronization parameter 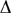, a varying number 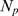 of cells is eligible to produce target waves. Since signal transduction (at the parameter setting used here) is enabled at 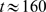 and the oscillatory regime starts at 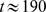 and ends at 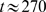 (cf. Figure 2), we can find this number by interpreting Eq. (7) as a function of 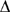,
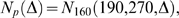
(1)which is a monotonically rising function for 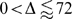. So, for 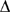 large enough to produce signaling patterns at all, and growing within reasonable bounds, one expects a growing number of potential pacemaker cells and hence a decreasing correlation between the (binary) spatial distribution of these cells and the time-averaged plane of detected target wave events because of the rising number of pacemaker cells that are entrained without leaving a strong fingerprint. Numerical experiments qualitatively confirmed this expectation: For 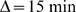, the correlation coefficient reaches about 40% and then decays rapidly with growing 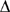 (data not shown).

**Figure 4:**
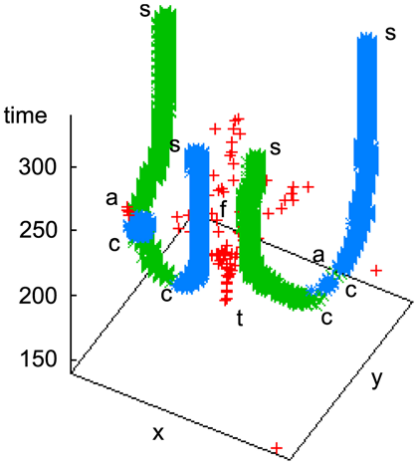
Space-time plot of the chain of events shown in Figure 3. This 3D representation reduces spiral waves tips and target wave origins to points, allowing one to see the whole temporal evolution at a glance. As before, target wave events are shown as red +, spirals as green (light gray)×(right handed) and blue (dark gray) * (left handed). Note that this picture represents only a single (but representative) realization of the system dynamics.

**Figure 5:**
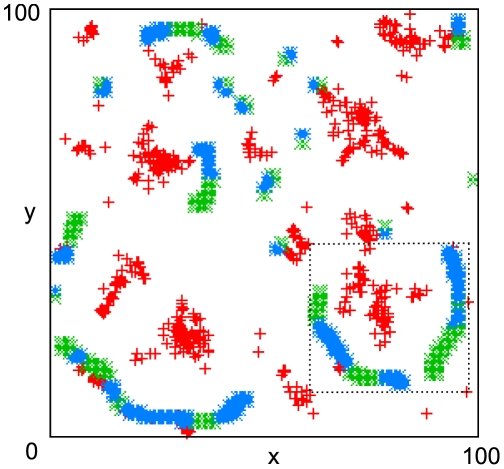
Space-time plot of the whole 100×100 grid shown in Figure 2 viewed from above. The predominantly transversal meandering of spiral tips around the pacemaker areas that created them is discernible. Target wave events are shown as red +, spirals as green×(right handed) and blue * (left handed), the dotted box indicates the area shown in Figures 3 and 4. The target wave events are the same as in Figure 1, viewed from above.

Combining the results for the detection of spiral- and target waves, we arrive at a fairly detailed picture of the behavior of the system (Figures 4 and 5). A typical repeatedly pulsing target wave (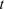) fractures (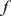) into several small active centers that drift apart in the oscillatory regime (see also Figure 1) and result in pairs of counter-rotating spirals (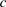) in an apparently typical distance from the original target wave center. The spirals in a pair repel each other (the target wave shrinks at both open wave ends, cf. [16]), drifting apart primarily transversally relative to the original target wave emitter and either annihilate with opposite-handedness spirals from an adjacent pair (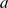, the wave segment vanishes) or come to rest and persist indefinitely in a dynamic steady state (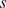). After spiral annihilation events, oscillatory cells in the vicinity can easily ‘hijack’ the location, since it has last experienced a very target-wave-like cAMP pulse, and initialize a new train of target waves (left 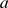, corresponding to the target wave visible in the lower left of Figure 3 at 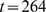); these persist at most until the last cells have left the oscillatory regime.

It is noteworthy that, comparing the raw system data with the phase data in Figure 3, phase singularities are often recognized by the algorithm long before the corresponding spirals in the raw data seem to come into existence; one could hence question the validity of this identification. However, the very continuous nature of subsequently identified phase singularities as shown in Figure 4, that in later stages also coincides perfectly with spirals apparent in the raw data, leads us to conclude that a creation of a pair of opposite-handedness phase singularities indeed constitutes the ‘birth’ of a counter-rotating spiral pair, even though it might not yet be discernible as such, looking only at the raw system data.

Do the sites of spiral pair creation events differ systematically from the rest of the cell population? We detected the sites of spiral pair creation events in many differently initialized runs of the model presented here, in search of another specific fraction of cells that is responsible for the breaking of target waves and hence for the creation of spiral wave pairs. To our astonishment, these events seem not be related with the position of a cell on the developmental path: The cell age offset values at the sites of spiral creation events exactly mimic the offset distribution, Eq. (5), of the whole population (data not shown). It thus seems that the locations of spiral pair creation are at least not directly influenced by cell properties, as we expected from looking into the previous works [3],[17].

**Figure 6:**
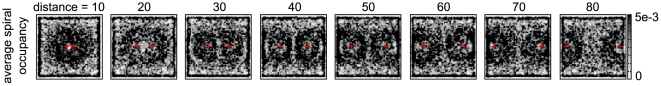
Temporal average of the spiral occupancy (disregarding handedness) for a pair of 3×3 pacemaker clusters (red +) in varying distances. Averages are over 500 minutes and 400 runs for each configuration. The cell positions on the developmental path were randomized in each run, except for the manually placed pacemaker clusters. The pattern revealed in these pictures cannot be extrapolated from single-run information as in Figures 4 and 5 (although the latter hints at it); it fully emerges only after an ensemble average is considered.

Is there a typical radius for spiral creation events, i.e. a typical distance from the target wave events around which the spiral waves organize themselves? We tested this hypothesis by running several hundred simulations in which the cell age distribution was randomized, but we placed 3×3 clusters of pacemaker cells (offset 60.0) at fixed positions before running the model. We chose completely synchronized clusters of pacemakers for simplicity, preempting their dynamical synchronization to the fastest pacemaker of the cluster, and at the same time allowing complete control over the relative phase of several pacemaker clusters. In fact, ring-like structures centered on the manually placed pacemaker clusters are visible in the average spiral wave occupancy (Figure 6), but their radius and crispness (i.e., the visual clarity) depend on the distance between the pacemaker clusters. For small distances up to 20 grid points, no separate rings are discernible and the localization of the ring is very poor, indicating a wide spread in spiral creation radii. For medium distances (30–60) there are clearly separated halos that are much more localized. For larger distances the appearance seems to revert to two separate instances of the weakly localized rings observed for very small distances.

**Figure 7:**
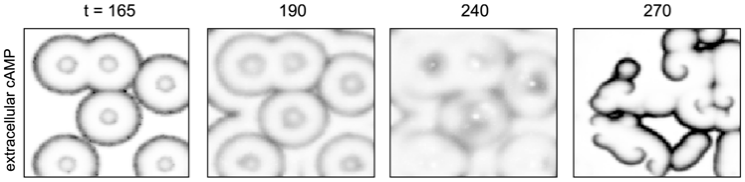
System snapshots of the extracellular cAMP concentration 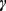 using a depleted cell age distribution with no randomly occurring pacemakers apart from those placed manually. Comparing this figure with Figure 2, the system's evolution is not qualitatively changed apart from the removal of ‘noise’ coming from random pacemaker candidates.

These effects can be explained in an artificially created minimal situation. In order to reduce the observed average patterns to contributions of the manually placed pacemaker clusters, we changed the cell age offset distribution, Eq. (5), to not contain random pacemaker cells. To achieve this, when creating the concrete cell age offset distribution for a given run, cells with an age offset between 20 and 110 minutes (corresponding to pacemaker candidates when the medium becomes active at about 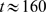, plus a head start of ten minutes for manually placed pacemakers to dominate the system) had their values re-randomized according to Eq. (5) until they ended up outside of this interval. In Figure 7 we demonstrate that this modified cell age distribution does not qualitatively change the temporal evolution of the system (apart from removing ‘noise’), and that the few manually placed pacemaker clusters are sufficient to generate the initial stage target waves as well as spiral waves later on.

**Figure 8:**
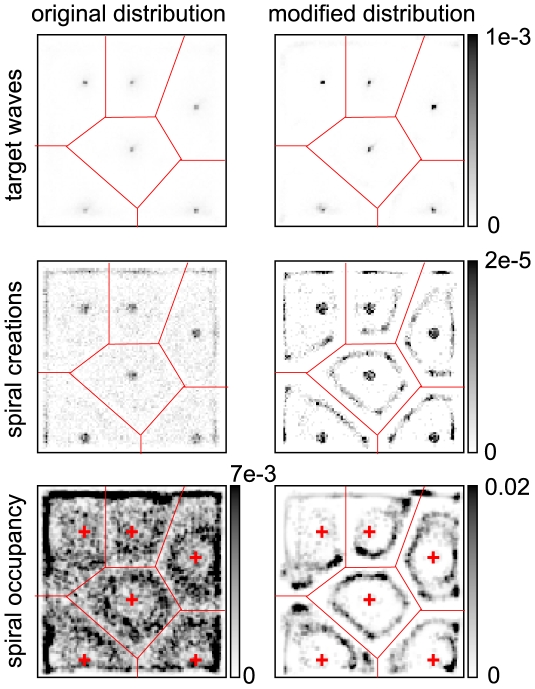
Comparison of the statistics of significant events using the original cell age offset distribution (left column) and the distribution depleted of random pacemakers described in the text. The top row shows the detected target waves clearly centered on the manually placed pacemaker clusters (as indicated in the bottom row) in both distributions. The center row shows the occurrence of spiral pair creation events; using the original cell age distribution, nothing much can be discerned, except for an artefactual bunching at the pacemaker locations. Using the depleted distribution on the right side reveals the close correspondence to the Voronoi diagram of the pacemaker clusters (red lines), showing increased amplitude near vertices and on direct connecting lines. The bottom row shows spiral occupancy, representing mainly the final steady state with fixed spirals. The contrast is drastically sharper using the depleted distribution (right column) due to the absence of ‘randomly’ emerging target waves. If the aspect ratio of a Voronoi cell is far from one, multiple halos can be formed, indicating that spirals in fact predominantly meander transversally to the pacemaker that created them.

The resulting spiral formation and -occupancy statistics of this modified cell age distribution, however, are vastly clearer than before (Figure 8). It is now readily discernible that the formation of spiral wave pairs (omitting boundary effects) happens almost exclusively on lines parallel to and in a fixed distance from the Voronoi diagram of the pacemaker clusters (i.e. the partition of the plane into areas that have the nearest pacemaker in common). Spiral pairs are formed in front of areas where waves collide and annihilate, probably based on a prolonged refractory time (or oscillation period) in these areas, due to the large peak amount of extracellular cAMP deposited there. In contrast to common expectation, we could not observe this process as a sudden breaking of target waves, it rather seems to be a gradual build-up of phase lag during the time when most cells are oscillatory (cf. 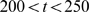 in Figure 3). The very exact localization near a specific distance from the Voronoi lines is due to the synchronization of our manually placed pacemakers – since their age offset is exactly identical, they fire in phase and hence the target waves emanating from pairs of adjacent pacemakers always meet exactly at half their distance to each other. Also, consistent with the reason for spiral formation stated above, the probability of spiral pair formation is higher near vertices of the Voronoi diagram, representing points where target waves from three or more pacemakers meet and annihilate, and on direct connections between adjacent pacemakers, where target waves collide head-on.

Removing the artificial constraint that all manually placed pacemaker clusters oscillate in phase does not qualitatively change this result. Since the pacemakers already are in the oscillatory regime when signaling is enabled, their phase difference can at most be 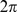, corresponding to one oscillation period 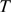. We selected the starting time offset of all pacemaker clusters uniformly from 60 to 66 minutes (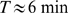). The phase difference distribution of adjacent pacemaker clusters is then triangular, peaking at zero and stretching to very unlikely maximum differences of 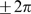. Hence, the positions of target wave collisions are still strongly centered on half the corresponding pacemaker cluster distances and the resulting distributions corresponding to Figure 8 are slightly washed out but qualitatively unchanged; the Voronoi diagram of the pacemaker clusters is still clearly discernible as the entity governing the statistics of spiral pair formation and consequently spiral occupation.

We hence conclude that the locations of spiral pair creation do not directly depend on the cellular properties at these sites, but on geometrical constraints which are an indirect consequence of the heterogeneity of cell properties given by their positions on the developmental path.

Note that the Voronoi diagram of the pacemaker cells arises here by the dynamical exploration of the ensemble of possible points for spiral formation over many numerical runs. It is not connected to the fact that in *D. discoideum* cell streaming experiments, the initially homogeneous plane is separated into several basins of attraction, which very nearly correspond to the Voronoi cells of the spiral cores (again, subject to phase differences). The Voronoi pattern in Figure 8 is a summary of the geometrical constraints arising implicitly from the distribution of cells on the developmental path, while the explicit partitioning into Voronoi cells during aggregation is simply a consequence of each *D. discoideum* cell moving to the nearest spiral core under the influence of the chemotactic signal.

The mechanism of spiral formation outlined above was not discernible in similar clarity, employing the unmodified cell age distribution, because of interference from randomly emerging pacemakers. The varying crispness in halo appearance in Figure 6 can also be explained in these terms. For very small distances, the two clusters effectively act as one pacemaker, since no fully formed waves are established between them, and spiral pairs are formed only by interactions with random pacemakers, which emerge at different positions, radii and relative phases in every numerical run, leading to a very fuzzy average image. Once the distance between the two manually placed clusters is large enough so that fully developed target waves are created emerging from each of them, this pair constitutes the most predominant (and consistent) cause of spiral formation, giving rise to fairly clear halos, especially directly between them. The further these clusters are separated, the higher is the probability of interference from random pacemakers, until at the maximum considered distance one effectively has two instances of a single consistent pacemaker cluster, interacting only with their respective varying neighborhoods of randomly emerging pacemakers.

Translating these insights back to the original setup of the model is straightforward in principle, but tricky in detail. Since for a desynchronization 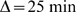 a large fraction (≈29%) of all cells has the potential to act as pacemaker cells, but few actually emerge because of the system's limited carrying capacity for sustained target waves per area, one needs a good scheme to predict the formation sites of initial target waves. The spatial density of pacemaker candidates is a promising start, but not quite sufficient, as Figure 1 shows. This difficulty can be reduced by choosing 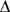 smaller, just large enough to give the randomly emerging pacemakers time to establish target waves before all cells enter the oscillatory regime, but the number of pacemaker candidates (e.g. ≈1350 for 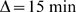 and a 100×100 grid, cf. Eq. (1)) is still substantially larger than the number of persistent target waves the system can sustain (few tens).

Alternatively and more realistically, regarding a possible application to experimental data, one can start from an ignorance of detailed cell properties and predict the spiral positions only from observed target wave positions. Since one cannot expect single experiments to yield statistically significant agreement with an ensemble average of many idealized repetitions we performed *in silico*, it might be most instructive to only compare observed spiral tip density per unit area in two categories, namely ‘high’ and ‘low’ expected spiral density, computed from the distribution of early target wave emitters. An experimental setup with a high degree of similarity to our manipulated cell age distribution might be attainable by placing a few (possibly fluorescence-marked) cells from an older population already in the target wave phase into a younger colony where target wave signaling has not yet been established. The geometrical spatial systematics may very well serve as an experimentally accessible evidence for such a developmental path in the real system.

### Comparison with experimental data

Other researchers have strived to explore the spatial correlations between target and spiral waves in experimental data [14], but so far the analyses proved difficult. We here for the first time apply methods from point process statistics [18] to the analysis of *Dictyostelium* signaling patterns. As outlined in the Methods section, this allows a quantification of the over- or underrepresentation of pairs of events at specific distances, resulting in typical ‘correlation profiles’ between target waves and spiral cores over distance. This method can at most very indirectly capture more detailed geometric correlation patterns (such as demonstrated in the previous Section), but allows the quantification of pair correlations in data sets that appear unordered to the naked eye. In addition, this method allows in principle to perform a cumulative analysis of many experimental runs, even despite their larger diversity when compared to numerical simulations.

We digitalized the data points in Figure 2a of [14] and compared the resulting curves to curves extracted from the model from [6] (‘Levine model’), the developmental path model analzyed here (with 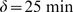, corresponding to 29% pacemaker candidates) and additional experimental data sets kindly provided by Christiane Hilgardt (University of Magdeburg) and Satoshi Sawai (University of Tokyo, [19]). We show here only one curve per (experimental) source, where the quality of target and spiral wave detection was highest. Apart from detection artifacts, all experimental curves exhibited the same qualitative behavior.

**Figure 9:**
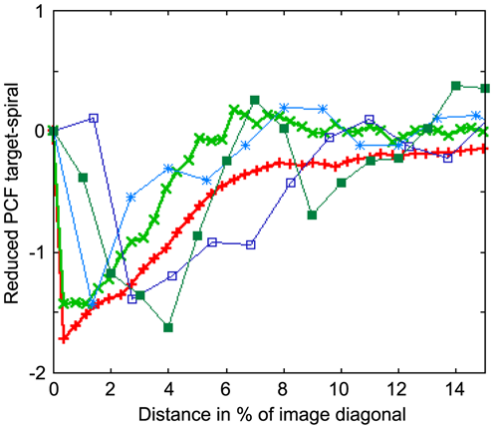
Reduced partial correlation function for target and spiral waves over distance in fraction of sample image diagonal. The curves shown are from the developmental path model (bold red +), the Levine model (bold light green ×) and experimental data scanned from LCG96 ([14], light blue *) as well as further experimental data kindly provided by Christiane Hilgardt (University of Magdeburg, dark blue squares) and Satoshi Sawai (University of Tokyo, dark green filled squares, [19]). All curves show a reduced probability to find pairs of spiral and target waves at very short distances, which corresponds to the anticorrelation we found numerically for the mathematical models. The Levine model shows this anticorrelation on a shorter spatial scale (recall that the grid constants differ by almost a factor 2 between the Levine and developmental path models, see Methods).

Figures 9–11 show the reduced partial pair correlation functions (see Methods) for target-spiral, spiral-spiral and target-target comparison, respectively.

**Figure 10 pcbi-1000422-g010:**
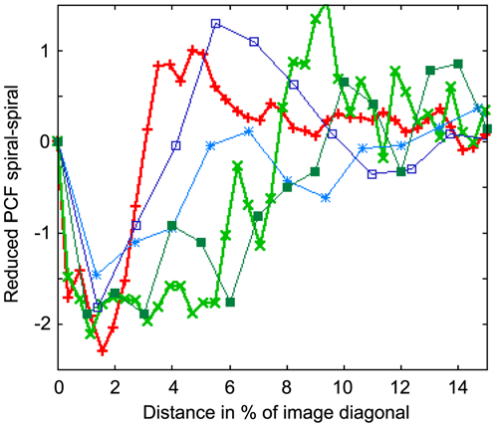
Reduced partial correlation function for pairs of spiral wave tips over distance in fraction of sample image diagonal. Colors as in Figure 9. All curves show a strong repulsion of spiral pairs for very short distances, followed by a range of overrepresentation in almost all curves, which we found to be mostly due to pairs of counterrotating spirals.

**Figure 11:**
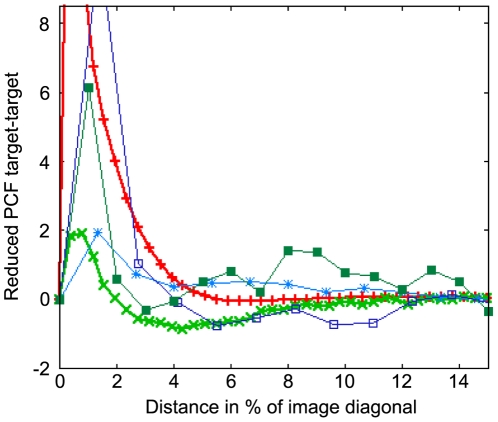
Reduced partial correlation function for pairs of target waves over distance in fraction of sample image diagonal. Colors as in Figure 9. All curves show an overrepresentation at very short distances, but the amplitude differs quite strongly between the mathematical models (the red curve for the developmental path model peaks at a value of above 20). The experimental data exhibit strongly varying degrees of local target wave repetitions.

The interplay between target and spiral waves (Figure 9) is dominated by an underrepresentation (suppression) of these pairs for short distances, which is the qualitative anticorrelation we found in [10] as well as in our present work. Note that all experimental curves we analyzed also exhibit this feature qualitatively. In some cases it can be somewhat obscured by crosstalk between target and spiral wave recognition, which is problematic mainly for experimental data sets (dark blue curve).

Figure 10 shows a strong suppression of spiral-spiral pairs below a minimum distance. This suppression typically has a much longer range than the minimum distance we manually imposed to prevent the double recognition of a single spiral signal as two points (the typical diameter of a spiral peak). Following this suppression there is a regime of overrepresentation that has its main root in the existence of stable pairs of counterrotating spirals. Note that this peak is apparently shifted towards higher distances for the model from [6], but this is mainly due to the smaller length scale this model is simulated at.

The most robust feature of Figure 11 is a significant overrepresentation of pairs of target waves in very short distances, indicating clustering. In the Levine model, this effect is much less distinct than in the developmental path model, due to the much smaller number of repeated firing events before a spiral pattern is established. The longer spatial scale in the developmental path model stems from the fracturing of target waves, resulting in large target wave clusters. One possible interpretation of this clustering is a correlation between the locations of specific ‘pacemaker’ cells and target waves (per construction, for the mathematical models). On the other hand, a clustering of target waves can also occur in scenarios where every quiescent cell has the potential to fire spontaneously (the original setup of [6]); the higher firing probability near the source of the previous target wave center is then based only on the longer time since the last firing event. The expected degree of clustering in such a setup depends on the typical time scale until newly quiescent cells fire, compared to the wave speed.

There is a possible objection to the anticorrelation hypothesis between spiral and target waves: One can argue that for the curves corresponding to spiral-spiral and spiral-target pairs there is a competition which trivially blocks these events from occuring in close proximity , whereas for the target-target curve there is no such competition since they occur sequentially instead of at the same time. This is absolutely true for the spiral-spiral case, we in fact assume that the length of the repulsive plateau is an indicator for the shortest length scale at which stable spiral pairs can coexist.

However, if one accepts that spiral waves in some form or other result from target waves, which are the fundamentally simpler patterns that can easily arise from spontaneous firing events of few cells, one has to accept that target waves first occur prior to spiral waves, when there is no coexistence and hence competition. This is rather clearly the case for both mathematical models considered here, were we observe a relatively sharp transition from target to spiral waves. The aforementioned objection hence only holds for target-spiral pairs in the late stage of signaling when spirals have been established. Furthermore, since both mathematical models considered so far qualitatively predict a target-spiral anticorrelation for short distances, this point does not contribute to the ultimate question of which model better captures the experimental evidence.

It should be noted that, ideally, one would analyze correlations between cell properties and pattern features. At the moment, however, to our knowledge such data do not exist for *Dictyostelium*. Exact length scales for experimental data would also be valuable in comparing several curves over real-space distances.

## Discussion

In this report we employed a technique for the identification of spatio-temporal target waves and used it in conjunction with spiral tip recognition, based on the established phase singularity technique, to identify typical temporal motifs of events in a developmental path model for the social amoeba *Dictyostelium discoideum*. This analysis follows up on our earlier investigation into the predictability of spiral patterns from the knowledge of cell properties conducted in a more schematic model of *D. discoideum* [10]. Not surprisingly, the more complex and more closely biologically motivated model examined here exhibits a more complex statistical dependence of the resulting spiral patterns on the cell properties. Nevertheless, we were able to identify a specific fraction of cells that function as effective pacemakers, as well as the dominant mechanism of spiral formation, and employ this knowledge to engineer the spatial statistics of target wave and spiral creation by manipulation of the pair correlations of these cells.

Similarly to our findings in [10], one observes an anti-correlation between (now dynamically generated, effective) pacemaker cell locations and the locations of spiral formation and asymptotic spiral position. However, the structure of spiral creation and meandering in the region between pacemaker locations is more complex; spiral tips are formed at a specific distance from lines of the Voronoi diagram of the pacemakers, and meander on roughly circular orbits around pacemakers, without intruding into the halos of adjacent pacemakers. Also, in strong contrast to the ‘simple’ anticorrelation we found in [10], the area near the lines of the Voronoi diagram (the area right between pacemakers) is expected to hold a strongly reduced amount of spiral tips.

The results presented here provide further evidence supporting our general hypothesis that single element properties are systematically mapped onto patterns and thus conserved through processes of self-organization (as opposed to enslaved and deleted by the collective), as outlined in [10] and the introduction of this paper. Furthermore, we have now presented mapping schemes for two numeric models of *D. discoideum*, yielding different predictions regarding the relationship between pacemaker positions and spiral wave tip statistics.

We compared both of these models to experimental data from [14], introducing the statistical tool of point processes to the *Dictyostelium* signaling debate. We were able to demonstrate that the spiral and target waves in the data from [14] are not uncorrelated, as claimed there, but that they follow qualitatively identical systematics as other experimental data as well as both considered mathematical models, including the anticorrelation between target wave centers and asymptotic spiral core positions. We have so far not been able to conclusively distinguish which mathematical model better captures the real system, mainly due to the increased noise level and the large variation one typically observes from run to run in experiments.

## Methods

### Computational model

The *D. discoideum* model considered here has been formulated in [20] and extended to include a developmental path in [5], with some additional discussion in [17]. It is given by three coupled differential equations for the total fraction of active cAMP receptor (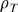) and the normalized concentrations of intracellular (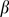) and extracellular (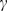) cAMP, respectively,
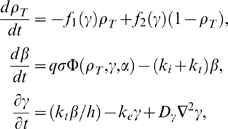
(2)with
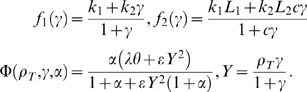
(3)The biological meaning of the main terms in these equations are the following: The cAMP receptors on the cell surface are de- and resensitized depending on the extracellular cAMP concentration and the currently active receptor fraction. Intracellular cAMP is produced depending (among other factors) on the current activity 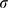 of the adenylate cyclase (cf. below). Upon diffusing to the extracellular area it is degraded by the action of phosphodiesterase (PDE, both bound to the cell membrane and extracellular PDE) and otherwise diffuses freely. The exact forms of the nonlinearities stem from the reduction from nine to three dynamic variables performed in [20].

For clarity, we use exactly the notation from [5]. This model has been studied in great detail in terms of its dynamical regimes as a function of the position in parameter space (cf. [20] and references therein). Here we do not consider a wide range of parameter constellations and instead focus on the dynamical processes leading to target and spiral wave formation. Throughout this paper we use the parameter setting discussed in [5], i.e. 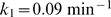, 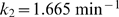, 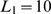, 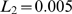, 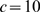, 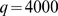, 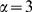, 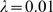, 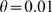, 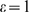, 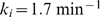, 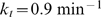, 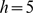, 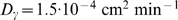. The grid spacing is 100 µm, a system time unit is identified with a minute [5]. We integrated these equations using an explicit Euler scheme with a fixed step size 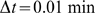, again as in [5]. The simulations were performed in custom software in C++.

**Figure 12:**
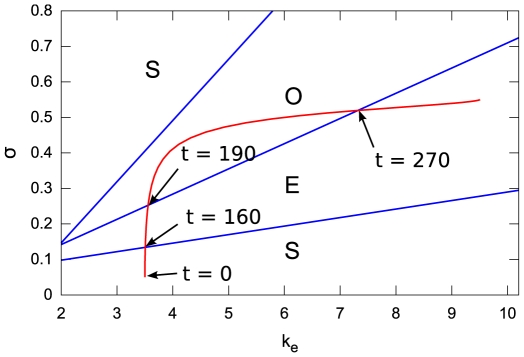
Developmental program imposed on the cells in a two-dimensional parameter plane given by the activities of adenylate cyclase in the cell and the rate of extracellular cAMP degradation caused by phosphodiesterase. Arrows with time indices indicate approximate passage times for cells with zero starting time offset. Cells start in the unexcitable (steady state) regime 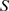 in the lower left area, then cross an excitable regime (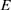) upwards into an autonomously oscillating regime (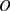), from where they move back into 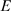 in the upper right area of phase space.

These equations and parameter settings give rise to several dynamic regimes, including most importantly a steady-state regime 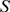 where external cAMP stimuli do not trigger a reaction, an excitable regime 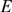 where external stimuli are followed by a sharp increase of cAMP production with a subsequent recovery period, and finally an oscillatory regime 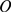 where the cells autonomously oscillate between phases of cAMP production and quiescence (Figure 12).

Lauzeral et al. [5] proposed that the maturation of cells has the effect of modifying their behavior (described in this model by the maximum activity of adenylate cyclase 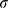 and the extracellular phosphodiesterase rate constant 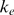), transporting them through these regimes along a fixed predetermined *developmental path*, thus giving rise to macroscopic cAMP patterns (cf. Figure 2) which then give the cue for cell aggregation and finally lead to the following stages of the cell cycle. In order to introduce the initial heterogeneity needed for the formation of spiral waves, it is assumed that cells have different properties at the onset of starvation, for example different stages of their cell cycle, which are represented as differing starting position offsets on this path.

Here we consider only the developmental path 3 of [5], given by a combined sigmoidal variation of 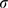 and 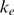,
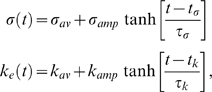
(4)where again we follow [5] closely, both in notation and in the parameter values, i.e. 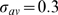, 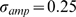, 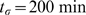, 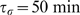, 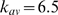, 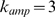, 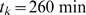, 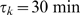. Figure 12 shows this developmental path in the parameter plane of a single oscillator (after [5]). A total number of 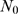 cells arranged on a regular spatial grid is placed on this path with varying starting time offsets 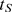 according to an exponential probability density,
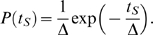
(5)Note that we refer to each grid point as a ‘cell’ for practical purposes, although it actually represents a cluster of about ten cells with synchronized properties [5]. Throughout this paper we use grids of 100×100 cells and 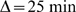 unless explicitly noted otherwise. It is noteworthy that the complete dynamics of this model depends only on the concrete choice of starting time offsets and contains no other random elements.

The exponential distribution implies that the number of cells with starting time in the interval 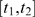 is approximately
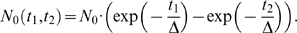
(6)After a time 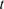 has passed, the number of cells in the interval 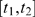 is
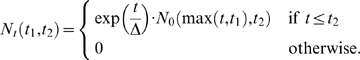
(7)


### Detection methods for spiral and target waves

We used algorithms to detect target and spiral wave configurations, which are well-known attractor states for excitable media dynamics, and which play important roles in the shaping of the self-organized cAMP communication process.

In order to detect spiral waves, we used the phase singularity method introduced by Gray et al. and Bray et al. [21],[22] in the context of heart tissue dynamics, which was to our knowledge first applied to *D. discoideum* data in [12]. We observed an influence of the sample size entering the specific average in the underlying embedding process (cf. [22]) on the exact recognized phase singularity position: Using a global average over all raw values (after the end of the quiescent period) for each grid point caused phase singularities of apparently pinned spiral waves to circle around an empty core on a decaying helix trajectory in space-time. While this did mimic the experimentally observed behavior, it was an inconvenience when trying to visually trace spiral cores. Using a gliding time average over about three signal periods (20 minutes) removed this meandering and yielded the spatially fixed phase singularities shown in this paper. Figure 3 shows a time series of raw system data contrasted with the corresponding phase data and detected phase singularities.

In order to detect target waves we developed a 3D fitting algorithm based on the already calculated smooth and amplitude-insensitive phase data (article in preparation). At its core, it fits cones (the spatio-temporal evolution of target waves, neglecting curvature effects on wave speed) to connected voxel segments, representing contour shells extracted from the spatio-temporal phase data and subjected to causal consistency constraints (maximum observed wave velocity). The tip of a successfully fitted cone corresponds to the point in space-time where an observed target wave was created; we call these points *target wave events*. An analogous spiral fitting algorithm was also developed, but not employed because of high computational cost and inferior performance compared to the phase singularity technique, given the relatively low-noise environment of the computational model discussed here.

### Point processes

We used the mathematical concept of *point processes* to quantify correlations between temporal projections of spiral and target wave events in the developmental path model [5] discussed here, the more phenomenological excitability model from [6] and experimental data from [14]. The core idea of point processes is to take a given distribution of possibly several types of points (*marked point processes*, here: locations of target wave events and asymptotic positions of spiral waves) and calculate a variety of measures comparing e.g. the observed frequency of point pairs in specific distances to the expected frequency if the points were randomly distributed (see e.g. [18]).

We found the *partial pair correlation function* to be the most distinct and at the same time straightforward quantifier of the relevant system statistics. For the two dimensional case used here it is defined as [18]

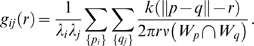
(8)Here 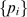 and 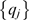 are the sets of all points of types 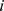 and 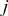, respectively, 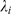 and 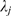 are the respective *intensities* (expected number of points of type 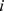 or 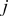 per unit area), 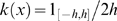 is the box kernel function with bandwidth 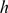 and 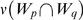 is the area of the intersection between the sampling window W shifted to 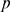 and 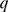. The latter term is intended to correct for edge effects. The bandwidth 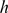 quantifies the width of the band around 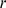 from which one accepts point pairs contributing to the value of 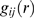 and should be chosen separately for each type of data set. Larger values increase the number of point pairs taken into consideration and thus improves the statistics but at the same time reduces the achievable resolution in 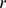; we increased 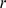 in steps of 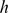. We used 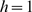 for data from numerical simulations, 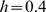 for the data from [14], 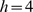 for the data by Christiane Hilgardt (300×500 pixel areas from digital photographies of dark-field experiments, downsampled to 150×250, to which the bandwidth refers) as well as for the data by Satoshi Sawai ([19], 640×480 downsampled to 320×200).

The partial pair correlation function quantifies the probability of simultaneously finding a point of type 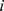 and another point of type 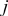 in infinitesimal volumes at distance 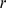, normalized by the expected probabilities. For complete spatial randomness one expects a constant value of one. Values greater than one indicate an overrepresentation of these pairs at distance 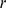 and correspondingly values of less than one correspond to underrepresentation. For large distances between points one expects an asymptotic behavior tending towards one, where the distances become so great that points do not influence each other significantly; by definition, 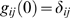.

Since the spatial resolutions and image sizes of the data we want to compare are different (and in some instances unknown), we renormalized distances to the maximum diameter of the sampling windows, i.e. the image diagonal. Length scales are then remapped to fractions of the image diameter and the curves become more easily comparable. One should keep in mind, though, that the 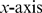 does not represent the same real-space distance for all curves, e.g. for the simulations we used identical 180×180 grids, but a single grid distance corresponds to 0.6 mm in the model from [6] (as used in [12]) and to 1.0 mm in the developmental path model analyzed in-depth here.

We found that edge effects are still visible despite the edge correction term, so we always plot 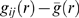, which we call the reduced partial pair correlation function, where 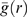 is the average 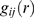 curve from hundred realizations of randomly distributed points in the same sampling window, keeping the numbers of target wave events and spirals fixed to the original amount found in the respective source data. Values above or below zero thus correspond to over- or underrepresentation compared to the null model of completely random point distributions, in units of the expected probability based on the intensities.

It should be clear that given the rather finite-sized sampling windows and the differences between the considered data sets, our comparisons based on this technique should be used mainly as qualitative indicators.
